# Rapidly Progressive IgA Nephropathy in a Patient With a History of Breast Cancer: A Case Report and Literature Review

**DOI:** 10.1155/crin/5546873

**Published:** 2026-05-25

**Authors:** Roger Leon Montesinos, Edwin Quispe Marca, Carla Arnez Torrico, Daniel Segura Herbas, Luis Céspedes Limachi, Zulma Tapia Arenas, Wilder Villca Mamani

**Affiliations:** ^1^ Department of Nephrology, Hospital de Clínicas, La Paz, Bolivia, hcpa.edu.br; ^2^ Department of Gastroenterology, Bolivian-Japanese Gastroenterology Institute, Cochabamba, Bolivia

**Keywords:** breast cancer, crescentic glomerulonephritis, IgA nephropathy, rapidly progressive glomerulonephritis

## Abstract

IgA nephropathy (IgAN), or Berger’s disease, is the most common primary glomerulopathy worldwide. It is characterized by dominant mesangial deposition of immunoglobulin A1 (IgA1), typically of the galactose‐deficient form (Gd‐IgA1), which triggers the formation of circulating immune complexes and subsequent glomerular inflammation. Clinical presentation includes hematuria, proteinuria, and, in severe cases, progressive renal failure. A rare variant is the extracapillary or crescentic form, which follows an aggressive course. We report the case of a 52‐year‐old woman with a history of Stage II right‐sided infiltrating ductal breast carcinoma, treated in 2023 with radical mastectomy, AC‐T chemotherapy, and radiotherapy. Complete oncologic remission was confirmed by SPECT imaging. In January 2025, with a known baseline creatinine of 1.2 mg/dL, the patient presented with macroscopic hematuria, hypertension, and rapid decline in renal function. Workup revealed subnephrotic proteinuria (1854 mg/24 h), active urinary sediment with 65% dysmorphic erythrocytes and red blood cell casts, and a creatinine peak of 4.7 mg/dL. Autoimmune serologies were negative. Kidney biopsy showed IgAN with cellular crescents in 64% of glomeruli and an Oxford classification of M0 E0 S1T1 C2. Immunofluorescence confirmed mesangial IgA and C3 deposits. Despite treatment with intravenous methylprednisolone, cyclophosphamide, and oral prednisone, yet no renal recovery was achieved, and the patient remains on chronic hemodialysis. To our knowledge, this is the first reported case in Bolivia of extracapillary IgAN occurring after breast cancer remission, highlighting a rare clinical association and raising the possibility of an underlying immune‐mediated link between malignancy and crescentic IgAN, without establishing causality.

## 1. Introduction

IgA nephropathy (IgAN) is the most common primary glomerulopathy worldwide, characterized by predominant mesangial deposition of immunoglobulin A in the glomeruli. Clinically, it typically presents with persistent microscopic hematuria and mild to moderate proteinuria. However, a small subset of cases may follow a more aggressive course, with rapidly progressive renal failure, subnephrotic or nephrotic‐range proteinuria, and crescent formation in glomeruli—referred to as crescentic IgAN [1, 2].

In this context, the presence of macroscopic hematuria along with rapidly deteriorating kidney function, as observed in severe forms of IgAN, represents a diagnostic and therapeutic challenge. These presentations are often associated with poor renal outcomes and limited recovery, even with timely and intensive immunosuppressive therapy [3, 4].

On the other hand, the association between glomerular diseases and solid tumors has garnered increasing interest in the literature. Paraneoplastic or immune‐mediated mechanisms have been proposed as potential triggers. While some glomerulopathies—such as membranous nephropathy—are more clearly associated with malignancies, the relationship between IgAN and solid tumors like breast carcinoma remains rare and poorly understood [5–7].

We present the case of a patient with a history of breast cancer in remission who developed crescentic glomerulonephritis due to IgAN, with macroscopic hematuria and rapidly progressive kidney dysfunction. To our knowledge, this is the first documented case in Bolivia with these features. Rather than establishing a causal relationship, this report aims to highlight a rare clinical scenario and discuss a possible temporal and immunologic association between malignancy and aggressive IgAN.

## 2. Case Presentation

A 52‐year‐old woman from Cobija, Bolivia, was diagnosed in 2023 with Stage II (T2N1M0) right‐sided infiltrating ductal breast carcinoma. Immunohistochemistry revealed hormone receptor positivity (estrogen and progesterone), HER2 negativity, and a Ki‐67 index of 30%, indicating moderate proliferative activity. She underwent a right modified radical mastectomy with axillary lymph node dissection (Levels I and II), followed by six cycles of adjuvant AC‐T chemotherapy (doxorubicin and cyclophosphamide followed by paclitaxel), and 30 sessions of external radiotherapy. Follow‐up imaging including SPECT showed no evidence of disease activity, and she was considered in complete remission under oncologic surveillance.

In 2024, she was diagnosed with systemic arterial hypertension and started treatment with losartan and amlodipine. In January 2025, she was admitted to the Boliviano Japonés Hospital in Cobija with a three‐week history of persistent macroscopic hematuria, dysuria, and bilateral lumbar pain. Initially diagnosed as a complicated urinary tract infection, she was treated with intravenous ciprofloxacin and analgesics. Her renal function at admission was normal (creatinine: 1.2 mg/dL).

However, her clinical symptoms persisted and progressed to nausea, vomiting, fatigue, loss of appetite, and bilateral lower limb edema. Serum creatinine rose rapidly to 4.7 mg/dL. She was referred to a tertiary care center in La Paz, where nephrology evaluation revealed a rapidly progressive nephritic syndrome. Urinalysis showed subnephrotic proteinuria (1854 mg/24 h), persistent macroscopic hematuria with > 200 red blood cells per high power field, and active sediment with 65% dysmorphic erythrocytes and red blood cell casts. Renal ultrasound showed early parenchymal nephropathy and a simple left renal cyst. Laboratory tests showed elevated uric acid (9.9 mg/dL) and mild hypokalemia (3.0 mmol/L); other parameters were within normal limits.

On physical exam, she had hypertension (172/95 mmHg) and lower limb edema. Immunological workup (antinuclear antibody [ANA], antiglomerular basement membrane [anti‐GBM], anti‐dsDNA, antineutrophil cytoplasmic antibody [ANCA]‐C, ANCA‐P, C3, and C4) was negative. Serum protein electrophoresis showed mild polyclonal hypergammaglobulinemia with decreased A/G ratio (1.0). No monoclonal bands were detected. Viral serologies for HIV, HBV, and HCV were negative. CRP was markedly elevated (51.2 mg/dL) (Table 1).

**TABLE 1:** Laboratory findings at admission.

Parameter	Result	Reference range/unit
White blood cells (WBCs)	8700	5000–10,000/mm^3^
Hemoglobin (Hb)	10.1	12.5–17 g/dL
Platelet count (Plt)	341,000	150,000–400,000/mm^3^
Total proteins	5	6.2–8.5 g/dL
Serum albumin (Alb)	3.8	3.5–5.1 g/dL
Serum creatinine	4.7	0.8–1.2 mg/dL
Urea	107	10–50 mg/dL
Sodium (Na^+^)	141	136–145 mmol/L
Potassium (K^+^)	3.3	3.5–5.0 mmol/L
Chloride (Cl^−^)	104	97–111 mmol/L
Phosphorus (P)	5.2	2.5–4.5 mg/dL
24 h proteinuria	1854	28–150 mg/24 h
Antinuclear antibodies (ANAs)	4	> 1:10 U/mL
HEp‐2 cells	5.9	> 10 U/mL
Anti‐DNA (ss)	22	< 99.0 IU/mL
Anti‐DNA (ds)	19.6	< 37.0 IU/mL
Complement C3	148	84–193 mg/dL
Complement C4	21.3	16–47 mg/dL
Anti‐GBM antibodies	Negative	< 1 AI
Rheumatoid factor	7.5	< 8.0 U/mL
C‐reactive protein (CRP)	51.2	< 1.35 mg/dL
p‐ANCA	0.034	Cutoff: 0.068
c‐ANCA	0.02	Cutoff: 0.114
HBsAg	0.037	Cutoff: 0.050
Anti‐HBc	0.139	Cutoff: 0.420
Anti‐HCV IgM	0.056	Cutoff: 0.200
Anti‐HCV IgG	0.097	Cutoff: 0.510
HIV 1/2	0.006	Cutoff: 0.180
Urinary RBCs	> 200/HPF	NA
Dysmorphic RBCs (%)	65	< 30%
Polymorphic RBCs (%)	1	< 5%
IgG	902	800–1600 mg/dL
IgA	375	70–400 mg/dL
IgM	120	90–180 mg/dL
Serum protein electrophoresis	No monoclonal band	NA
Urinary immunofixation	No monoclonal band	NA

*Note:* Hb: hemoglobin; Plt: platelet count; Alb: albumin; ANA: antinuclear antibodies; HBsAg: hepatitis B surface antigen; HBc: hepatitis B core antigen; IgA: immunoglobulin A; IgG: immunoglobulin G; IgM: immunoglobulin M; C3: complement component 3; C4: complement component 4.

Abbreviations: AI, antibody index; ANCA, antineutrophil cytoplasmic antibodies; CRP, C‐reactive protein; dsDNA, double‐stranded DNA; GBM, glomerular basement membrane; HCV, hepatitis C virus; HIV, human immunodeficiency virus; HPF, high power field; RBCs, red blood cells; ssDNA, single‐stranded DNA; WBC, white blood cells.

Given the suspicion of immune complex‐mediated rapidly progressive glomerulonephritis, she received three pulses of intravenous methylprednisolone (1 g/day). Due to persistent uremic symptoms and worsening azotemia, she was started on hemodialysis. A renal biopsy (Figures 1 and 2) revealed 14 glomeruli with 64% cellular crescents and 14% fibrocellular crescents. No global sclerosis was present. There was Bowman’s capsule rupture, extracapillary proliferation, mixed interstitial inflammatory infiltrate (10%), 20% interstitial fibrosis, and 10% tubular atrophy. Oxford score: M0 E0 S1 T1 C2. Immunofluorescence showed mesangial deposits of IgA (++), C3 (+), kappa (+), lambda (+), and IgG (+), with negative IgM and C1q. Fibrinogen was positive in extracapillary proliferative areas.

FIGURE 1Light microscopy of renal biopsy at × 400 magnification. (a) Hematoxylin and eosin (H&E) stain showing a glomerulus with a prominent cellular crescent and endocapillary hypercellularity. (b) Periodic acid–Schiff (PAS) stain highlighting extracapillary proliferation and disruption of the glomerular basement membrane. (c) Jones methenamine silver stain revealing a well‐formed cellular crescent with preserved capillary loops and focal basement membrane irregularities.

**Figure:**
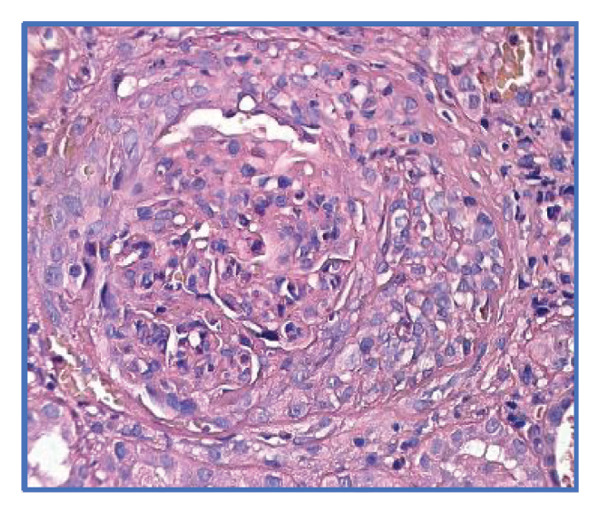
(a)

**Figure:**
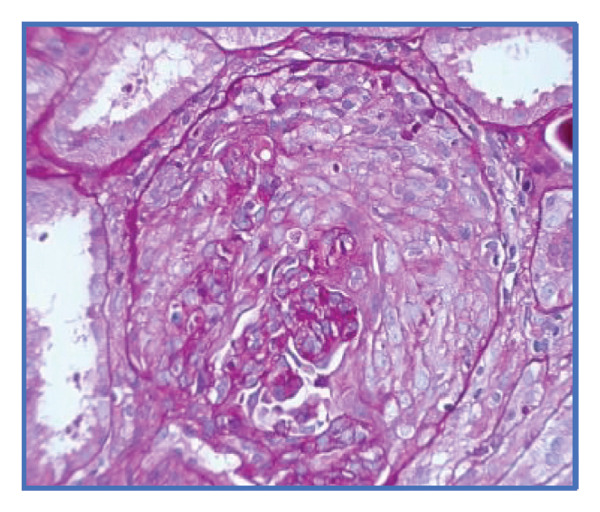
(b)

**Figure:**
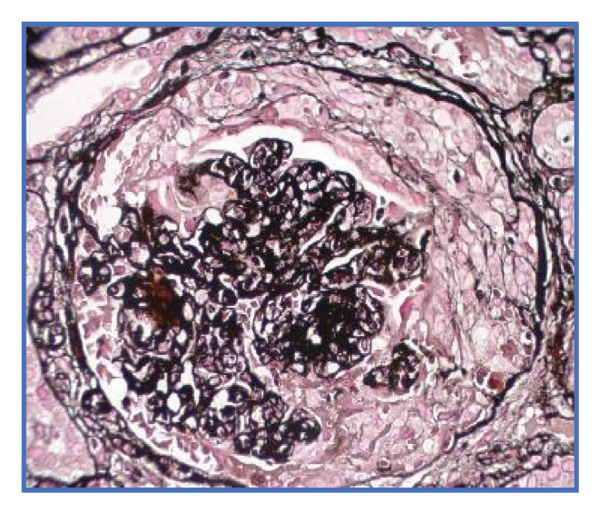
(c)

FIGURE 2Direct immunofluorescence on fresh frozen tissue at × 400 magnification. (a) Mesangial deposits of IgA with intensity 3 (+). (b) Fibrinogen deposits in extracapillary proliferative areas corresponding to cellular crescents.

**Figure:**
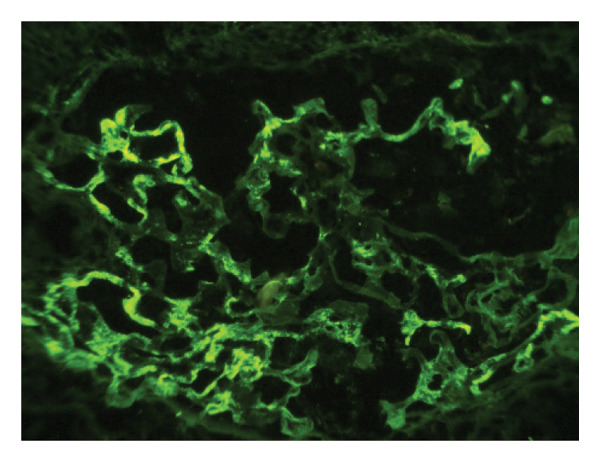
(a)

**Figure:**
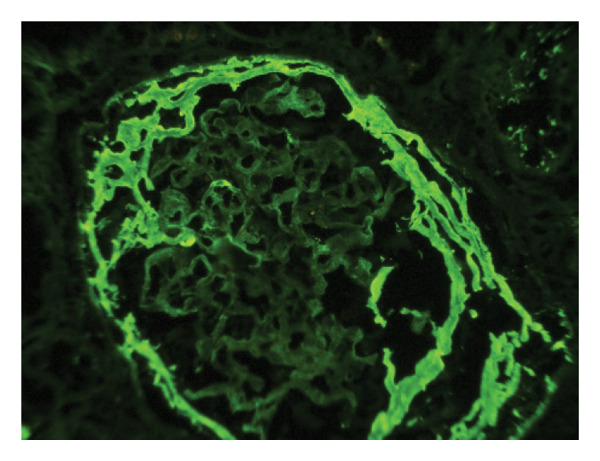
(b)

Immunosuppressive therapy with six pulses of intravenous cyclophosphamide (500 mg every 15 days) and oral prednisone (1 mg/kg/day) was initiated, with prophylaxis using acyclovir and trimethoprim–sulfamethoxazole. During treatment, the patient developed catheter‐related bacteremia due to *Staphylococcus epidermidis*, which was resolved with targeted antibiotics. Despite completing immunosuppression, there was no renal recovery, and the patient remains on chronic hemodialysis.

## 3. Discussion

IgAN is characterized by mesangial deposition of galactose‐deficient IgA1 (Gd‐IgA1) and may present with recurrent hematuria, proteinuria, and hypertension. While the disease course is typically indolent, a minority of patients develop a rapidly progressive form with crescent formation, also referred to as extracapillary IgAN or rapidly progressive glomerulonephritis, which is associated with poor renal outcomes [8, 9].

The diagnosis of primary IgAN is established based on kidney biopsy findings demonstrating dominant or codominant mesangial IgA deposition on immunofluorescence, in the appropriate clinical context. Importantly, this diagnosis requires exclusion of secondary causes of IgA deposition, including chronic liver disease, infections, autoimmune disorders, and malignancy‐associated processes. In our patient, negative autoimmune serologies, normal complement levels, negative viral markers, and absence of monoclonal bands on serum and urine protein electrophoresis supported a primary glomerular process.

The differential diagnosis of crescentic IgAN includes other causes of rapidly progressive glomerulonephritis. The main entities to consider are ANCA‐associated vasculitis, antiglomerular basement membrane disease, and lupus nephritis. These conditions were excluded in our patient based on negative serologic markers, including ANCAs, anti‐GBM antibodies, and ANAs, as well as normal complement levels.

In addition, infection‐related glomerulonephritis and monoclonal gammopathy–associated renal disease were considered unlikely given negative viral serologies and the absence of monoclonal bands on serum and urine protein electrophoresis. Histopathological findings, particularly dominant mesangial IgA deposition on immunofluorescence, supported the diagnosis of IgAN.

This case describes severe extracapillary IgAN in a patient with a history of breast cancer in remission. Her subacute presentation included persistent gross hematuria, rapid renal function decline, active urinary sediment, and dialysis requirement within weeks. The biopsy revealed extensive crescent formation (> 60% of glomeruli), classifying the case as aggressive IgAN, which occurs in fewer than 5% of the patients and is associated with a high risk of end‐stage kidney disease [10].

We acknowledge that a direct causal relationship between breast cancer and IgAN cannot be established based on a single case. Crescentic IgAN may occur as a primary disease and could have been incidentally identified in a cancer survivor. However, the temporal proximity to cancer remission, the aggressive histological pattern, and the absence of secondary causes raise the possibility of an immune‐mediated association rather than a proven paraneoplastic mechanism.

Although associations between glomerulopathies and malignancy are well documented—particularly membranous nephropathy—the link between IgAN and solid tumors remains rare and poorly understood. Isolated reports have described IgAN in association with gastric, pulmonary, renal, and breast cancers, but these observations remain insufficient to infer causality [11–13].

Cancer or its treatment may promote aberrant IgA1 production or immune complex formation capable of mesangial deposition. Chemotherapeutic agents like anthracyclines may alter mucosal or mesangial immune tolerance, potentially unmasking latent autoimmune disease [14]. In this patient, no infections or recent immunomodulatory treatments were identified, supporting a unique and isolated pathogenic event.

Serum IgA levels were not available in this case. However, it is important to note that serum IgA levels are neither sensitive nor specific for the diagnosis of IgAN and are not required for diagnosis, which relies primarily on histopathological findings.

Serum protein electrophoresis showed polyclonal hypergammaglobulinemia and a low albumin–globulin ratio, consistent with chronic immune activation seen in glomerulopathies, without monoclonal gammopathy [15].

Despite aggressive immunosuppressive therapy with corticosteroids and cyclophosphamide, renal function did not recover. The presence of > 50% crescents, interstitial fibrosis, and tubular atrophy are known predictors of poor prognosis, even with timely treatment [16].

A preliminary version of this work was previously presented as a poster at the World Congress of Nephrology and published as an abstract in Kidney International Reports [17]. The current manuscript represents a substantially expanded and original version, including additional clinical data, detailed histopathological findings, and a more comprehensive discussion.

This report has important limitations. First, it represents a single observational case and, therefore, cannot establish causality between breast cancer and IgAN. Second, no specific biomarkers or immunologic studies were available to confirm a paraneoplastic mechanism. Finally, the possibility that crescentic IgAN developed as a coincidental primary glomerular disease in a cancer survivor cannot be excluded. Nevertheless, the temporal association, the unusually aggressive presentation, and the exclusion of secondary causes justify reporting this case as a rare clinical scenario.

## 4. Conclusion

We describe a rare and aggressive case of crescentic IgAN in a patient with a history of breast cancer in remission. Although a temporal association was observed, a direct causal relationship between malignancy and IgAN cannot be established from a single case, and the disease may have occurred as a coincidental primary glomerulopathy.

Nevertheless, the unusually rapid clinical course, extensive crescent formation, and exclusion of secondary causes raise the possibility of an underlying immune‐mediated association rather than a proven paraneoplastic mechanism.

This case highlights the importance of evaluating oncology patients with hematuria or acute renal dysfunction for glomerular disease, even during remission, and underscores the need for further studies to clarify this rare clinical association.

## Funding

This research received no specific grant from any funding agency in the public, commercial, or not‐for‐profit sectors.

## Conflicts of Interest

The authors declare no conflicts of interest.

## Data Availability

The data that support the findings of this study are available from the corresponding author upon reasonable request.
